# Understanding the Male Perspective: Evaluating Quality of Life and Psychological Distress in Serbian Men Undergoing Infertility Treatment

**DOI:** 10.3390/life13091894

**Published:** 2023-09-11

**Authors:** Bojan Čegar, Sandra Šipetić Grujičić, Jovana Bjekić, Aleksandar Vuksanović, Nebojša Bojanić, Daniela Bartolović, Darko Jovanović, Milica Zeković

**Affiliations:** 1 Clinic of Urology, University Clinical Center of Serbia, 11000 Belgrade, Serbia; bojancegar@gmail.com (B.Č.); avuksano@open.telekom.rs (A.V.); bojanicnebojsa@gmail.com (N.B.); darkojov@gmail.com (D.J.); 2 Faculty of Medicine, University of Belgrade, 11000 Belgrade, Serbia; 3 Institute of Epidemiology, Faculty of Medicine, University of Belgrade, 11000 Belgrade, Serbia; sandra.sipetic-grujicic@med.bg.ac.rs; 4 Human Neuroscience Group, Institute for Medical Research, National Institute of Republic of Serbia, University of Belgrade, 11000 Belgrade, Serbia; jovana.bjekic@imi.bg.ac.rs; 5 Center for Medical Biochemistry, University Clinical Center of Serbia, 11000 Belgrade, Serbia; daniela_pejak@hotmail.com; 6 Centre of Research Excellence in Nutrition and Metabolism, Institute for Medical Research, National Institute of Republic of Serbia, University of Belgrade, 11000 Belgrade, Serbia

**Keywords:** male infertility, health-related quality of life, semen examination, psycho-emotional distress

## Abstract

The experience of an infertility diagnosis and treatment imposes a profound burden on affected individuals, encompassing not only physical and medical aspects but also a plethora of psychological, social, and emotional factors. By employing a multimodal assessment featuring validated self-report questionnaires, physical measurements, and clinical records, the present study aimed to explore the quality of life and psycho-emotional distress of men undergoing infertility treatment in Serbia, thereby addressing the dearth of research on the underrepresented male perspective in this domain. Findings revealed diverse semen abnormalities among participants (n = 96, average age 37.69 ± 5.72), with significant associations between longer treatment durations and reduced sperm motility. The observed rates of men surpassing predetermined DASS-42 questionnaire thresholds for depression, anxiety, and stress in the analyzed cohort were 13.54%, 11.46%, and 22.92%, respectively. Summary scores in conceptual areas comprised in the SF-36 questionnaire ranged from 49.00 ± 6.25 for the mental health dimension to 90.16 ± 17.75 obtained in the physical functioning subscale. Patients with a longer treatment duration demonstrated lower scores in the role emotional domain, indicative of a less favorable emotional state. Expectedly, inverse correlations were found between the SF-36 mental health score and DASS-42 subscales. By addressing the existing knowledge gap and highlighting the unique needs of infertile men, the finding of this study may contribute to a more inclusive and holistic approach to infertility research and management.

## 1. Introduction

Infertility represents a major reproductive health issue with substantial clinical, humanistic, and economic burdens, alongside demographic repercussions. It is defined as a disease of the female or male reproductive system characterized by the failure of achieving clinical pregnancy after engaging in regular unprotected sexual intercourse for 12 months or more [1,2]. With the staggering global lifetime prevalence reaching approximately 17.6% of the adult population, based on World Health Organization (WHO) estimates for 2022, infertility may have a profound and devastating impact on contemporary society and affected individuals [2]. The magnitude of this public health concern, hindering the attainment of several United Nations Sustainable Development Goals (SDGs), underscores the need for a comprehensive and multifaceted agenda targeting policy formulation and advocacy, research efforts, and educational activities concomitantly with the provision of reliable, accessible, and equitable fertility care services [3]. Addressing the infertility in a responsible and coordinated manner is of paramount importance for fostering sexual and reproductive health and rights and, subsequently, the overall physical and mental wellbeing. 

The intricate and heterogeneous pathogenic landscape of infertility challenges both its epidemiologic and causative analysis. Although the interaction between partners determines the fecundity, it is estimated that pure or combined male-factor-associated infertility is present in approximately 50% of all the couples experiencing undesired childlessness [4]. The etiology of male infertility encompasses a broad spectrum of pre-testicular causes, testicular disorders, and post-testicular conditions. The underlying mechanisms may be traced to disrupted spermatogenesis, gonadal and extragonadal endocrine disorders, congenital or acquired anatomical defects, functional urogenital anomalies, immunological and genetic-related causes, sexual dysfunction, infections, certain chronic illnesses, environmental exposures, and lifestyle determinants [5,6]. Nevertheless, in 30–40% of primary testicular failure subjects, the etiology remains elusive, and these cases are referred to as idiopathic male infertility [7]. 

Despite reasonable limitations regarding the assessment of the overall spermatozoal fertilizing capacity, intraindividual variability in relevant parameters over the course of time, and analytical standardization issues, conventional semen analysis remains fundamental and often the most informative component of the infertility evaluation in men [8]. Nevertheless, quantitative and qualitative semen parameters complying with reference values do not necessarily equate to normal fertility, as some men may have latent health impairments or fertility disturbances. Conversely, abnormal semen analysis results do not unavoidably indicate infertility [9]. All men facing conception difficulties should receive a comprehensive medical assessment to identify and address any modifiable risk factors compromising their reproductive potential. The implementation of a structured multidisciplinary clinical care algorithm featuring reproductive endocrinologists, andrologists, and general urologists supports the streamlining of the diagnostic process and treatment planning, eventually resulting in better procedure coordination and enhanced patient care. By leveraging the expertise of these specialists, a collaborative practice model fostering a holistic and personalized approach establishes a dynamic platform for a seamless and well-orchestrated exchange of knowledge, insights, and resources, thereby facilitating the pursuit of targeted and tailored interventions, leading to superior patient outcomes. Furthermore, accumulating scientific evidence suggest that infertile men may have increased susceptibility to cardiovascular diseases, certain oncopathologies, and phycho-emotional disorders, underscoring the importance of appropriate screening and counseling [10,11].

The inability to meet personal desires and public expectations of male procreation is often recognized as a threat to the traditional perception of masculinity and may be related to social stigma and significant impacts on the quality of life (QoL) of men and their partners [12]. In men who regard fatherhood as an important element of their male identity, the inability to become biological parents may trigger profound psychological distress featuring an array of negative emotions, including the feelings of shame, guilt, and inadequacy. By acknowledging the fertility issue and engaging in activities related to treatment, some men are forced to deconstruct and re-frame the embodied notions of their manhood [13]. Regardless of the rising prevalence trends in male factor infertility, this topic largely remains taboo, at least partially due to the societal norms discouraging men from disclosing their struggles and openly discussing their vulnerabilities. Furthermore, there is a notable gender imbalance in the current literature on infertility, as most studies have focused on the female position, thus often leaving the male perspective overlooked or underestimated [14,15]. Hence, it is essential to expand the breadth and depth of the scientific knowledge base regarding the QoL of infertile men to gain a deeper insight into their needs and to inform the development of effective interventions aimed at enhancing their overall health and welfare [16]. 

The objective of this study was to investigate the QoL and emotional distress of men undergoing infertility treatment in Serbia, using a multi-dimensional approach featuring validated self-report questionnaires, physical measurements, and medical records, to provide a holistic perspective of the complex interplay between psychological and physical factors in men’s experience of infertility. Particular emphasis was placed on the exploration of the impact of the treatment duration on the QoL and psycho-emotional disturbances, as well as the sequence of relationships between these constructs.

## 2. Materials and Methods

### 2.1. Study Design and Participants

This observational, cross-sectional study recruited a sample of patients attending the Clinic of Urology, University Clinical Center of Serbia for infertility treatment, from January to May 2018. The recruitment protocol included four consecutive stages: (1) identifying potential participants and screening against the predefined criteria, (2) approaching the selected men and proposing their inclusion in the current research project, (3) providing all the relevant information regarding the purpose of the study, responsible entities, and a detailed description of anticipated activities, and specifying the protection procedures applied for ensuring personal data privacy and confidentiality, prior to finally (4) seeking ethically valid and scientifically appropriate consent.

In order to limit participants’ burden and avoid additional clinical appointments that may compromise the overall recruitment rate, both data and sample collection were organized within the individual’s standard outpatient care schedule. All the recruitment activities were performed by trained healthcare professionals, i.e., 4 responsible urologists serving as members of the core research team. Acknowledging the possibility of intentional and/or unintentional bias introduced by so-called “gatekeepers” (research mediators arbitrating the suitability of subjects to access the potential participants’ pool) [17,18], the selection protocol and conditions were clearly articulated and formally approved by the internal Clinic’s Professional Board, and the initial screening stage was performed concomitantly by two independent researchers. 

Eligible participants were (1) adult men of 18 years and older willing to join the study, (2) experiencing at least one year of primary or secondary male-factor-associated infertility confirmed by a specialist in accordance with The European Association of Urology (EAU) Guidelines on Male Infertility [19], with an (3) absence of cognitive disability precluding the capacity to provide responses to study questionnaires independently and accurately. The exclusion criteria were as follows: (1) compromising mental illnesses or severe functional impairments, (2) a history of alcohol and/or drug abuse that may distort the validity of the obtained results, (3) present psychiatric disorders requiring treatment, and (4) limited language/communication capacities.

A total of 118 men undergoing infertility treatment were approached over the course of the recruitment process and screened against the predefined eligibility criteria. Among suitable subjects, 102 voluntarily agreed to participate in the study. Nevertheless, due to failure to adhere to the research protocol and provide all the required data prior to study completion, 4 men were disqualified and thus excluded from further analysis. Furthermore, 2 decided to withdraw their consent, yielding a final sample of 96 male individuals (average age 37.69 ± 5.72, range: 21.00–52.00 years) and an overall response rate of 81.36%.

### 2.2. Ethics Statement

This non-incentivized study was conducted in accordance with the ethical principles and standards of medical research involving human subjects expressed in the Declaration of Helsinki. The protocol was approved by the Ethics Committee, Faculty of Medicine, University of Belgrade (approval no. 29/XII-7). All research participants provided written informed consent for data collection, semen sample provision, and the subsequent analysis. 

### 2.3. Data Collection

#### 2.3.1. General and Medical History Data

Participants’ sociodemographic, lifestyle, and medical data were collected with a purposefully devised questionnaire in a standardized manner via structured face-to-face interviews conducted by trained healthcare professionals. By maintaining a professional and objective attitude, the interviewers aimed to establish an atmosphere of trust, thus ensuring that each subject was treated respectfully and with dignity. General information included age, residential region, education, occupation, prior paternity, and self-perceived socioeconomic status. The compilation and presentation of the highest attained level of formal education were performed in compliance with the well-established International Standard Classification of Education (ISCED) reference framework [20], whereas the patients’ professional profile was indicated based on categories proposed by The International Classification of Occupations (ISCO) [21]. Furthermore, the questionnaire addressed relevant lifestyle determinants (i.e., smoking and alcohol consumption) and diverse environmental factors associated with an adverse impact on male fertility (exposure to extreme ambient temperature, pesticides, solvents, and chemical toxins) [22]. Comprehensive medical data, including present acute and chronic conditions, past medical and surgical history, and family history, were retrieved from personal health records. 

#### 2.3.2. Anthropometric Assessment

The anthropometric assessment was performed in a clinical setting during the physical examination by skilled medical staff. Participants’ body weight in light clothing and without footwear was measured using an electronic platform scale with tarring capacity calibrated to 0.1 kg (Tanita BC-545N, Tanita Corporation, Tokyo, Japan). Height was recorded with an accuracy of 0.1 cm with a mobile stadiometer (Seca Leicester Portable Height Measure; Seca GmbH & Co KG, Hamburg, Germany). Body Mass Index (BMI) was calculated as a ratio of weight and standing height squared (kg/m^2^). Based on BMI, subjects were allocated to normal weight (18.50 kg/m^2^ ≤ BMI ≤ 24.90 kg/m^2^), overweight (25.00 kg/m^2^ ≤ BMI < 30.00 kg/m^2^), and obese categories (BMI ≥ 30.00 kg/m^2^) [23]. 

#### 2.3.3. Physical Activity Evaluation

Patients’ physical activity estimates were based on the internationally acknowledged and broadly applied self-administered International Physical Activity Questionnaire—Short Form (IPAQ-SF) [24]. The instrument referring to the 7-day recall period captures vigorous and moderate-intensity physical activities, walking, and sitting, all undertaken across a wide-ranging set of domains, including occupation-related activities, transportation, domestic/household chores, and leisure time. Featuring seven short (numerical) response items, the IPAQ-SF imposes minimal respondents’ burden, thus implying negligible survey fatigue-induced bias [25]. Following the questionnaire scoring manual and the Compendium of Physical Activities coding scheme [26], the activity scores were converted into metabolic equivalents (METs) in minutes per week. Such a measure, introduced to facilitate and promote the inter-study comparability of coded physical activity indices, is presented in both absolute values (total and intensity-specific contribution), as well as in the form of 3-level categorical scores (low, moderate and high activity). The sedentary period was not included in energy expenditure calculations, but is presented as a separate informative entity. The threshold for excessive sitting was set on 540 min per day [27]. 

#### 2.3.4. Evaluation of Psycho-Emotional Disturbance

The participants’ level of psycho-emotional distress was evaluated with the Depression Anxiety Stress Scales (DASS)-42 questionnaire [28]. This survey tool represents the tripartite construct characterized by a low positive effect, anhedonia, life devaluation, avolition, inertia, and hopelessness specific to depression, physiological hyperarousal, apprehension, and general distress pertinent to anxiety, and persistent irritability, impatience, agitation, nervous tension, and chronic non-specific arousal corresponding to stress [29]. A substantial research corpus featuring DASS-42 provides robust evidential verification of the questionnaire’s good psychometric properties, reliability, and validity in both community and clinical populations. DASS-42 encompasses forty-two self-report items divided into three subscales (fourteen statements each) referring to distinguishable, yet moderately inter-correlated, negative emotional symptom clusters (DASS-Depression, DASS-Anxiety, and DASS-Stress) [30]. The instrument’s reference period is the past week, and each item is rated on a 4-point Likert-type severity/frequency scale ranging from 0 to 3. Domain-specific scores are summed for the respective scales, with higher scores indicating more pronounced distress and a higher prevalence of the syndrome-related symptoms. Given that DASS-42 is based on the underlying assumption that emotional disorders intrinsically display a continuum of severity, this tool provides quantitative rather than categorical measures. Without the ambition to assign respondents to discrete diagnostic entities postulated by the conventional morbid taxonomic classificatory frameworks, DASS-42 scoring and interpretation guidelines propose cut-off values for percentile scores defining mild (78–87)/moderate (87–95)/severe (95–98)/extremely severe (98–100) labeled categories for each subscale.

#### 2.3.5. Health-Related Quality of Life Assessment

The 36-Item Short Form Survey (SF-36) was employed for the assessment of the health-related quality of life [31]. Accommodating the diversity of major dimensions and operational definitions of health and acknowledging the potential and utility of well-constructed standardized short-form assessment tools, SF-36 is a patient-reported generic multi-faceted eight-scale instrument. The comprehensive conceptual structure of the questionnaire encompasses the following spectrum of health domains: physical functioning, role physical, bodily pain, general health, vitality, social functioning, role emotional, and mental health. Following the introductory tool familiarization and filling out instructions, the questionnaire was self-administered in the presence of research staff. No proxies were allowed by the study protocol, and the completion time averaged 10–15 min. In order to minimize the desirability bias and interviewer-related confounding effects, researchers adhered to a standardized script aiming to communicate with respondents in a neutral, non-suggestive non-judgmental manner. 

The computational analysis of the SF-36 was performed as a multi-step procedure resulting in eight subscale-specific numerical scores. First, the pre-defined Likert-type values were re-coded and linearly transformed per the weighted percentage-based scoring key. Subsequently, items pertaining to the respective health domain were averaged to create eight summary measures ranging from 0 to 100, whereby lower values represent less favorable outcomes and more intensely perceived disability.

### 2.4. Semen Examination

Semen processing and analysis were performed by an accredited laboratory following standardized procedures compliant with the internationally acknowledged World Health Organization manual [32]. Ejaculates were obtained through masturbation after 3–7 days of sexual abstinence in a private room on clinical premises into a specialized sterile graduated wide-mouthed test-vessel made of spermatozoa non-toxic material. Men were provided with a detailed information sheet and precise spoken instructions concerning the appropriate protocol for semen sample collection. In order to prevent the adverse impact of sample exposure to temperature fluctuations and ensure a proper assessment of liquefaction, the specimen containers were placed in an incubator preset at 37 °C within 5 min after the collection. Routine macroscopic evaluation, volume determination, and preparation of dilutions and smears for the assessment of quantifiable features (sperm count and concentration) and spermatozoa qualitative attributes (i.e., their motility and morphology) were performed in the following 30–60 min. Patients were allocated to appropriate categories based on semen parameters. The lower reference limits for the semen volume, sperm concentration, total spermatozoa motility, and progressive motility were 1.5 mL, 15 × 10^6^ spermatozoa per mL of ejaculate, 40%, and 32%, respectively. Patients presenting with a reduced sperm concentration were further classified into 3 subgroups: severe (<5 × 10^6^ spermatozoa/mL of ejaculate), moderate (5–10 × 10^6^ spermatozoa/mL of ejaculate), and mild (10–15 × 10^6^ spermatozoa/mL of ejaculate) oligozoospermia. Sperm motility findings not meeting the defined criteria were considered asthenozoospermia. Patients with less than 4% of morphologically normal spermatozoa forms according to the Papanicolaou staining procedure and an observation with brightfield optics in oil immersion at 1000× magnification were diagnosed with teratozoospermia. The estimated presence of leukocytes determined by the peroxidase activity assay in concentrations exceeding the consensus-based threshold value of 1.0 × 10^6^ per mL of ejaculate was labeled as pyospermia.

### 2.5. Statistical Analysis 

Statistical analysis was performed using IBM SPSS Statistics for Windows, Version 28.0. (IBM Corp. Released 2021, Armonk, NY, USA), and open-source JASP software, version 0.17.2 (University of Amsterdam, Amsterdam, The Netherlands) [33]. Continuous data were summarized with the appropriate measures of the central tendency (mean and median values) complemented by measures of dispersion (standard deviation and the interquartile range), whereas categorical data were presented in absolute numbers and frequencies. The normality of the variable distribution was explored with the Kolmogorov–Smirnov test. Differences between sample subgroups were assessed using the Mann–Whitney U test or the Chi-square test, in accordance with the variable type. Spearman’s rank correlation was employed to evaluate the strength and direction of associations between DASS-42 and SF-36 scale scores. In conjunction with the conventional frequentist statistical approach, Bayesian inference was adopted with an aim to enhance the effect of the robustness estimation and credibility of the conclusions. Therefore, alongside frequentist statistical metrics, the Bayes factor (BF_10_) is reported as a quantitative indicator of the relative predictive performance of the two rival hypotheses. Such a hybrid approach fosters a more comprehensive and rigorous analysis, enabling well-informed and reliable interpretations of study findings while acknowledging and managing the inherent advantages and disadvantages of each statistical paradigm. Deviation from BF_10_ = 1, which indicates equal support for the null (H_0_) and the alternative hypothesis (H_1_), represents the degree of evidence in favor of either H_0_ or H_1_. Consensually, BF_10_ values greater than 1 are supportive of H_1_ (1–3: anecdotal; 3–10: moderate; >10 strong), while a BF_10_ lower than 1 favors H_0_ (1–0.33: anecdotal; 0.33–0.10: moderate; <0.10 strong) [34,35]. The mediation analysis was conducted in order to elucidate the role of depression, anxiety, and stress in the relationship between the SF-36 role emotional (SF-RE) domain and infertility parameters, i.e., the sperm concentration category and the duration of infertility treatment. Specifically, we aimed to assess whether the effect of infertility on SF-RE was direct or mediated by the levels of psycho-emotional disturbances.

## 3. Results

The overview of the descriptive characteristics of enrolled patients, stratified by treatment duration, is summarized in Table 1. With the average height, weight, and BMI of 181.58 ± 7.03 cm, 92.65 ± 18.01 kg, and 27.72 ± 4.01 kg/m^2^, respectively, the majority (n = 70, 72.92%) were overweight or obese, whereas the rest were allocated to the normal weight category. Respondents were predominantly employed (n = 94, 97.92%) residing in the urban setting of the capital city region (n = 79, 82.29%), childless (n = 82, 85.42%), and had self-perceived middle-level socio-economic status (n = 75, 78.13%). Current habitual cigarette and alcohol consumption was present in 29.17% and 56.25% of the studied samples, respectively. 

According to the IPAQ-SF assessment, three-quarters of the studied men were categorized as active and the cohort-level estimated total energy expenditure was 4262.59 ± 4425.25 MET-minutes per week. On average, the contributions of vigorous, moderate-level intensity, and walking activities were 1711.25 ± 2777.18, 1068.54 ± 1615.75, and 1482.77 ± 1395.10 MET-minutes per week, respectively. The self-reported daily sedentary period featured a broad range from 30 to 960 min and reached the average value of 374.12 ± 188.24 min. Excessive sedentary behavior, i.e., sitting for 540 min or more per working day was recorded for 15 men (15.62% total sample). Although not reaching the statistical significance threshold, subjects allocated to the low physical activity category had a higher BMI compared to the moderate and high activity groups (28.05 ± 3.46 kg/m^2^ vs. 27.80 ± 3.94 kg/m^2^ and 27.51 ± 4.37 kg/m^2^). 

Among respondents, fewer than 10% reported repetitive and prolonged exposure to occupational and environmental factors with a postulated detrimental impact on fertility (extreme ambient temperature: n = 7, 7.29%; pesticides: n = 2, 2.08%; solvents: n = 7, 7.29%; chemical toxins: n = 5, 5.21%). A review of personal medical records revealed a history of sexually transmitted infections in 13 patients (13.52%), whereas 9 (9.38%) were previously treated for non-gonococcal urethritis. In total, 15 participants (15.63%) had prior urological surgery, including inguinal hernia repair (n = 7, 7.29%), and varicocelectomy (n = 5, 5.21%). The overall burden of chronic non-malignant diseases was relatively low, with hypertension being the most prevalent condition (n = 19, 19.79%), followed by allergies (n = 12, 12.50%), diabetes (n = 4, 4.17%), and respiratory diseases (n = 4, 4.17%). In addition, four (4.17%) subjects were cancer survivors. Based on participants’ report, six (6.25%) had a family history of male infertility.

In the analyzed cohort, the infertility treatment duration ranged from 12 to 180 months, with an overall average of 34.80 ± 33.61 months. The reported period pertains to the time dedicated to diagnostic and therapeutic procedures aimed at achieving successful pregnancy. Treatment modalities included a spectrum of the available armamentarium, including the promotion of lifestyle modifications, sexual dysfunction management, treatment of urogenital tract infections, targeted endocrinologic interventions, surgical procedures and, most commonly, application of assisted reproductive technology treatments. Patients may have undergone different procedures either concomitantly or one after another, as the treatment evolved over time based on individual circumstances. Surgical approaches may be categorized into four main groups: interventions aimed at enhancing semen parameters (varicocelectomy), surgeries to optimize sperm delivery (namely transurethral resection of ejaculatory ducts in the case of obstruction), procedures for diagnostic purposes (testicular biopsy), and interventions for the retrieval of sperm specifically for in vitro fertilization (testicular sperm extraction, TESE). No statistically significant differences were observed between the subgroups of patients treated for less or more than two years (n_1_ = 50, n_2_ = 46, respectively) regarding the anthropometric indices, self-reported lifestyle determinants (alcohol, cigarettes, and coffee consumption habits), physical activity, prior urological pathologies (all *p* > 0.05, Table 1), and the general comorbidity burden, including allergies (*p* = 0.883) and cardiovascular (*p* = 0.941), respiratory (*p* = 0.941), hematological (*p* = 0.965), gastrointestinal (*p* = 0.286), and rheumatic diseases (*p* = 0.652), neurological and psychological disorders (*p* = 0.965), dermatologic conditions (*p* = 0.145), endocrine (*p* = 0.519) and metabolic disturbances (*p* = 0.523), hepatitis (*p* = 0.618), and cancer (*p* = 0.941).

Semen analysis revealed the complete absence of spermatozoa in the ejaculate of 10 (10.42%) patients. Sperm concentrations below the WHO lower reference limit, either isolated or present in conjunction with motility and/or morphology-related spermatozoa deviations, were found in 45 (46.87%) subjects. It is noteworthy that the majority of patients presenting with oligozoospermia had a severe form. The concomitant existence of multiple semen abnormalities, i.e., oligoasthenozoospermia, asthenoteratozoospermia, oligoteratozoospermia, and oligoasthenoteratozoospermia, was detected in 25 (26.04%), 27 (28.12%), 31 (32.29%), and 23 (23.96%) men, respectively. Poor sperm motility was more pronounced among active smokers (*p* < 0.001) and men experiencing fertility issues for a longer period of time. Accordingly, among individuals with infertility treatment exceeding two years, there was a significantly higher prevalence of asthenozoospermia (χ^2^(1, n = 96) 12.667, *p* < 0.001), oligoasthenozoospermia (χ^2^(1, n = 96) 10.682, *p* = 0.001), asthenoteratozoospermia (χ^2^(1, n = 96) 10.299, *p* = 0.001), and oligoasthenoteratozoospermia (*χ^2^*(1, n = 96) 11.159, *p* < 0.001). Teratozoospermia was detected in 33 (34.37%) subjects and was always associated with other seminal alterations. Fewer than 10% of the study participants had an abnormally elevated concentration of leukocytes in semen samples, and the majority (i.e., 79 (82.26%)) had an ejaculate volume within the reference range (Table 2).

**Figure 1:**
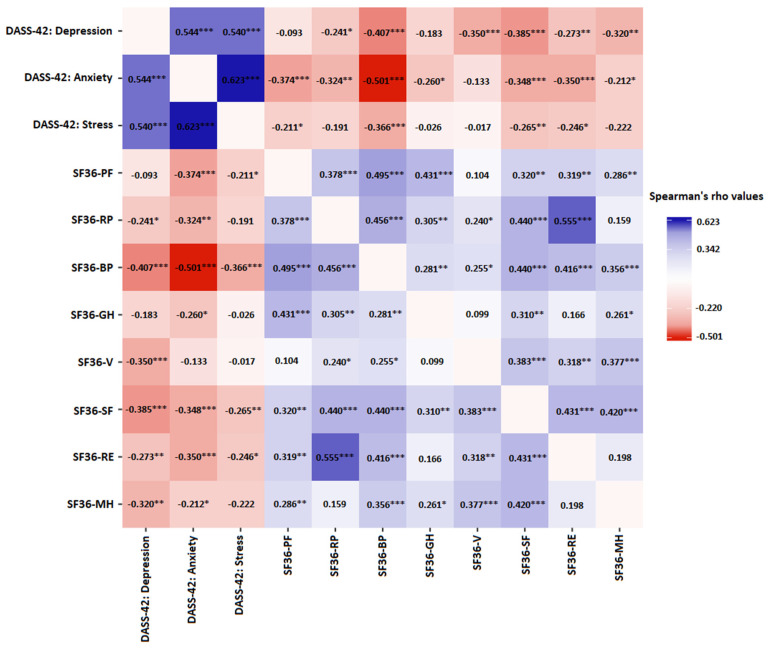
Heatmap correlation matrix presenting associations between The Depression Anxiety Stress Scale (DASS-42) and 36-Item Short Form Health Survey questionnaire (SF-36) domains. PF-physical functioning, RP-role physical, BP-bodily pain, GH-general health, V-vitality, SF-social functioning, RE-role emotional, MH-mental health; * *p* < 0.05; ** *p* < 0.01; *** *p* < 0.001. The colors used in the heatmap are designed to convey specific information about the correlations: red indicates a negative correlation, blue represents a positive correlation between two variables, while the color intensity (both red and blue) reflects the magnitude of the correlation coefficient. A stronger correlation, whether positive or negative, is depicted by a darker and more saturated color, while weaker correlations are shown with lighter shades.

The overall average scores based on the DASS-42 psychosocial distress evaluation were 4.52 ± 4.98, 3.89 ± 4.15, and 10.78 ± 7.83 points for the depression, anxiety, and stress negative emotional dimensions, respectively. A visual inspection of the distribution plots revealed right (positive) skewness for all the subscales, and the significant deviation from the normal distribution was confirmed through Kolmogorov–Smirnov tests. A summary of the obtained results featuring each scale central tendency and variability measures, conservative frequentist probability, and Bayesian inference metrics, along with further sample stratification based on conventional ratings from mild to extreme severity, are provided in Table 3. As presented, score discrepancies between patients undergoing infertility treatment for less or more than two years were not statistically significant based on a Mann–Whitney U test with the Bayes factors indicating moderate strength of evidence in favor of the null hypothesis for the anxiety (B_10_ = 0.244) and stress (B_10_ = 0.261) domains and weak (B_10_ = 0.582) for the depression subscale. Although it is important to reiterate that particulate domain scores should be primarily regarded as dimensional rather than categorical, the prevalence of subjects surpassing a priori defined cut-off points for depression, anxiety, and stress was 13 (13.54%), 11 (11.46%), and 22 (22.92%), respectively, confirming the established pattern of an absence of statistically significant differences between men treated for less or more than two years (depression subscale: χ^2^(1, n = 96) 1.118, *p* = 0.290; anxiety subscale: χ^2^(1, n = 96) 2.122, *p* = 0.145; and stress subscale: χ^2^(1, n = 96) 0.050, *p* = 0.824). Strong positive correlations between subscales corroborated conceptually related underlying constructs (Figure 1, all *p* < 0.001 and all Bayes factors B_10_ > 100).

Score distributions across eight health-related quality of life conceptual areas comprised in the SF-36 questionnaire are presented in Table 4. Component summary scores ranged from 49.00 ± 6.25 for the mental health dimension to 90.16 ± 17.75 obtained in the physical functioning subscale. Expectedly, correlations between scales were positive and, with a limited number of exceptions, statistically significant (Figure 1). The ceiling effect (defined as ≥15% of respondents reaching the highest possible score) was observed in physical functioning, role physical, and social functioning domains. When assessed against the infertility treatment period (<2 years and ≥2 years), the between-group difference reached the threshold value of statistical significance only for the role emotional concept. Patients with a longer treatment duration scored lower in this health domain indicating a less favorable state. A significant correlation with the negative direction and moderate magnitude was confirmed with the analysis featuring the treatment period as the continuous variable (Spearman’s ρ = −0.196, *p* = 0.05; Bayes factor (BF_10_) = 0.270). IPAQ-SF-based estimates of physical activity, expressed as MET-minutes per week, correlated inversely with the bodily pain subscale (Spearman’s ρ = −0.228, *p* = 0.05; Bayes factor (BF_10_) = 1.361). Patients presenting with (single or multiple) semen abnormalities did not differ from their normospermic counterparts regarding the results attained in any of the SF-36 health dimensions. Not surprisingly, significant positive correlations were observed between the scales encompassed by DASS-42, with Depression vs. Anxiety showing Spearman’s ρ = 0.544, Depression vs. Stress with Spearman’s ρ = 0.540, and Anxiety vs. Stress exhibiting Spearman’s ρ = 0.623 (all *p* < 0.001), while inverse associations were found between DASS-42 scores and SF-36 health domains. A heatmap plot, as the graphical representation of the correlation matrix indicating both direction and magnitude of the observed associations between these instruments, is presented in Figure 1.

**Figure 2:**
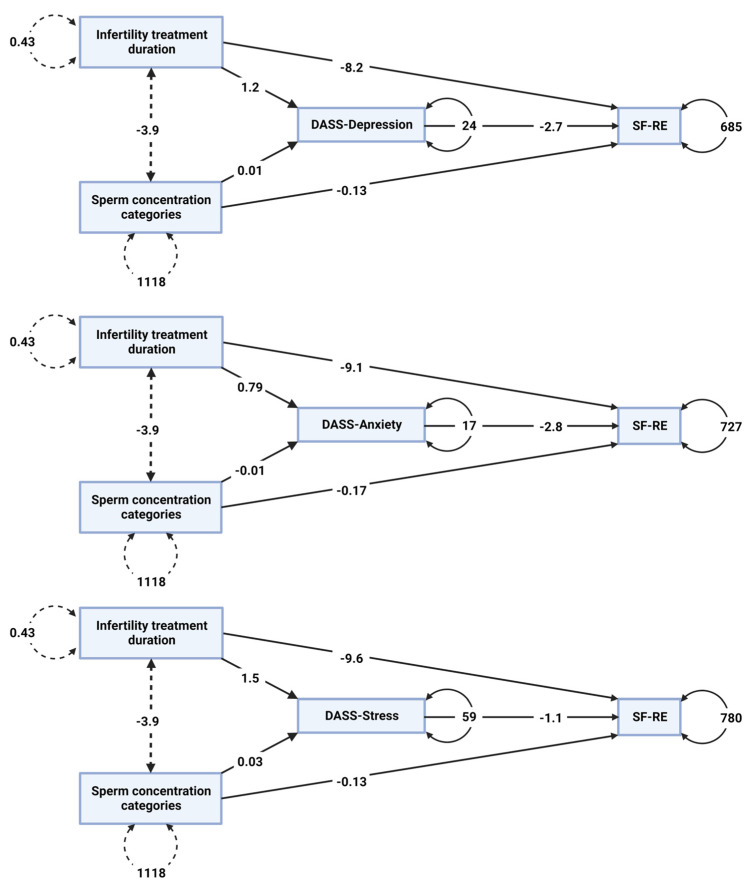
Mediation analysis path plots presenting direct, indirect, and total effects of infertility parameters (sperm concentration category and the duration of infertility treatment) on the role emotional domain of the quality of life assessed based on the SF-36 questionnaire (SF-RE) featuring psychoemotional disturbance (in the form of depression, anxiety, and stress subscale scores derived from DASS-42) as intervening variables; DASS-Anxiety: significant direct effects observed for the sperm concentration (*p* = 0.034) and treatment duration (*p* = 0.037) based on SF-RE, with treatment duration showing significant indirect effects (*p* = 0.244, *p* = 0.033); DASS-Depression: marginal significance for sperm concentration (*p* = 0.05) as a direct effect, no significant indirect effects observed (*p* = 0.154, *p* = 0.431); DASS-Stress: significant direct effect of the sperm concentration (*p* = 0.032) based on SF-RE, no significant indirect effects observed.

Individually constructed mediation models for depression, anxiety, and stress (as presented in Figure 2) entailed estimations of both direct and indirect effects. The mediation model pertaining to the DASS-Anxiety symptom cluster indicated a statistically significant direct effect of both the sperm concentration grouping (*p* = 0.034) and the time-extent of the infertility treatment (*p* = 0.037) based on SF-RE, while significant indirect effects were observed only for the treatment duration entity (*p* = 0.244, *p* = 0.033, respectively). The mediation model for DASS-Depression yielded less-convincing results, with direct effects demonstrating marginal significance for sperm concentration categories (*p* = 0.05) and no significant effects for the treatment duration (*p* = 0.123). Additionally, the model did not reveal any significant indirect effects (*p* = 0.154, *p* = 0.431, respectively). Similarly, the mediation model for the DASS-Stress cluster yielded results with only the direct effects of statistically significant levels for the sperm concentration class (*p* = 0.032). The direct effects for the infertility period, as well as indirect, i.e., mediation effects, were all bellow the statistical significance threshold. Total effects of the sperm concentration category and infertility duration based on SF-RE were both significant (*p* = 0.015, *p* = 0.032, respectively). This implies that SF-RE is directly affected by the explored infertility parameters and that the effect of the treatment duration is partially mediated by socioemotional distress expressed in the form of anxiety. 

## 4. Discussion

The experience of infertility diagnosis and treatment imposes a complex and multifaceted burden on affected individuals, encompassing not only physical and medical aspects but also a plethora of psychological, social, and emotional factors. Involuntary childlessness may instigate a broad spectrum of negative feelings, including guilt, embarrassment, reduced self-esteem, grief, anxiety, and depression, along with strained interpersonal relationships and social isolation [36]. Furthermore, the impact of infertility may extend beyond the personal domain and affect the couple’s bond, sexual functioning, and communication [4,37]. The current literature on infertility displays considerable gender asymmetry, with a predominant focus placed on the female position [38,39]. Thus, a more comprehensive and gender-inclusive approach in the evaluation and management of infertility-associated distress and its underlying determinants is essential to promote the overall well-being and QoL of those struggling with conception. By employing a multimodal assessment featuring validated self-report questionnaires, physical measurements, and clinical records, the present study sought to shed light on a nuanced interrelationship between physical and psychological factors affecting infertile men in Serbia, thereby addressing the dearth of research on the underrepresented male perspective in this domain.

The investigation yielded diverse semen abnormalities in the study cohort, with notable associations between a prolonged treatment duration and reduced sperm motility. Among the participants, 13.54% surpassed predetermined thresholds for depression based on the DASS-42 questionnaire, while 11.46% and 22.92% experienced elevated anxiety and stress levels, respectively. The summary scores from the SF-36 questionnaire demonstrated a broad spectrum, ranging from 49.00 ± 6.25 for the mental health dimension to 90.16 ± 17.75 for the physical functioning subscale. Additionally, patients with longer treatment durations exhibited lower scores in the role emotional domain, indicating a less favorable emotional state. As expected, inverse correlations were observed between the SF-36 mental health score and the DASS-42 subscales.

Although occasionally supplemented by advanced and more sophisticated sperm function tests, the routine analysis of semen, exploring the vital parameters, such as the concentration, motility, and morphology, retains its indispensable role as a cardinal procedure in the assessment and treatment of male partners in couples experiencing infertility. Accordingly, in conjunction with a thorough investigation of the reproductive and general medical history and rigorous hormonal and physical examinations, physiologically aberrant spermatozoa remain the primary target of meticulous scrutiny throughout the infertility management [40]. Within the study sample, a remarkable proportion of subjects was observed to manifest severe male-factor infertility, encompassing the conditions of severe oligospermia and azoospermia [41]. As advancements in the comprehensive evaluation of male reproductive function and diagnostic methodologies continue to unfold, these conditions remain a formidable challenge in the realm of infertility treatment [42]. The complete absence of spermatozoa was found in the ejaculate of 10 patients, constituting 10.42% of the study cohort. These findings corroborate the current epidemiologic estimates of azoospermia, indicating a prevalence of approximately 1% among the general male population and notably higher, ranging from 10% to 15%, among individuals diagnosed with infertility [43]. Historically, men diagnosed with azoospermia were categorized as unambiguously infertile, with sperm donation being the primary consideration for achieving parenthood. However, contemporary medical literature and practice have significantly advanced the understanding of azoospermia, revealing that many underlying causes of this condition are potentially reversible, thus offering new options as prospective avenues for restoring reproductive potential [44]. The advancement of knowledge and subsequent paradigm shift in the management of severe male-factor infertility hold the potential of positively influencing the overall QoL experienced by affected individuals. This biomedical evolution not only contributes to the improvement of their psychological well-being, but also acts as a catalyst in alleviating feelings of despair and cultivating a more positive and empowered perspective as they navigate the intricacies associated with confronting such health challenges [45]. This is echoed by the findings of the present study given that no significant differences were observed regarding the overall health-related QoL or psychosocial well-being between azoospermic or oligospermic participants compared to their normospermic counterparts, as measured by the SF-36 and DASS-42, respectively.

With accumulating evidence at the confluence of urology and reproductive biology, a general understanding has evolved to recognize that the functional capacity of spermatozoa extends beyond their mere fertilization potential, but rather includes their ability to orchestrate a normal course of embryonic development via diverse genetic and epigenetic mechanisms. These sperm-borne imprints are influenced by multiple paternal variables, such as the genetic makeup, advancing age, and certain modifiable risk-factors [46]. One of the most significant phenomena with adverse effects on sperm quality features is the excessive generation of reactive oxygen species (ROS), which may be related to a range of pathologic conditions, environmental exposures, and lifestyle determinants [47]. Asthenozoospermia, present in approximately one-third of study participants, can arise from structural abnormalities or functional impairments of spermatozoa, as well as the deleterious effects of seminal plasma or due to the synergistic interplay of these detrimental factors [48]. Apart from intrinsic causes, such as protein structural defects and genetic disorders contributing to deficiencies in sperm motility, certain physiological processes occurring over the course of sperm maturation and ejaculation may also exert effects via intricate molecular alterations and cellular signaling events. Perturbation of these occurrences and their intensification beyond regulated levels lead to the exacerbation of negative impacts on sperm movement capacity [49]. Within our sample, cigarette smoking emerged as a particularly prominent deleterious contributor among factors associated with oxidative stress and exposure to harmful chemicals, evidenced by the significantly higher prevalence of asthenozoospermia observed among active smokers. These findings are aligned with prior research, including a comprehensive meta-analytical review that summarized published evidence regarding the adverse impact of smoking on semen parameters derived from more than 5000 men using the WHO criteria [50]. The present study revealed a concerning observation regarding the excess weight prevalence among participants, given that the significant majority, exceeding two-thirds, were classified as overweight or obese. A potential association between the escalating incidence of infertility and obesity at a global level has generated substantial interest and apprehension within both the scientific community and public health sectors [51]. Extensive research has been conducted to explore the impact of the detrimental synergy between an increasingly sedentary lifestyle and an unfavorable dietary regimen prevalent in Western societies on the declining reproductive potential among males over the past half-century [52]. The impact of obesity on semen quality and the reproductive–endocrine milieu has yielded conflicting findings and remains a topic of debate and uncertainty in the scientific literature [53,54]. To attain more objective insights on this issue, it has been proposed that the research focus should be (re)directed towards ordinary obese men, rather than infertile individuals, thus mitigating the potential confounding factors present in these patients [55]. Our findings underscore the need for clinicians’ awareness regarding both the direct and indirect effects of obesity on fertility, a deeper understanding of the implicated physiological mechanisms and emotional disturbances, and their dedication to the implementation of tailored interventions. While additional investigations are required to fully ascertain the extent of efficacy and the precise role of each recommendation, it is essential to motivate individuals encountering fertility difficulties to maintain a healthy body weight, optimize their dietary habits, restrict alcohol consumption, engage in regular moderate-intensity physical activity, and cease smoking [38].

It is recognized that the experience of involuntary childlessness may exert adverse psychological effects, potentially giving rise to a paranormative crisis that detrimentally impacts men’s self-esteem, occupational functioning, and personal relationships, consequently heightening the likelihood of concomitant symptoms of anxiety and depression [56]. The observed rates of men surpassing predetermined DASS-42 thresholds for depression, anxiety, and stress in the analyzed cohort were 13.54%, 11.46%, and 22.92%, respectively. When contrasting our findings with existing literature, it is worth noting that the documented occurrence of psychological symptoms among infertile men exhibits considerable variation across diverse investigations. A Slovenian study involving 353 infertile men attending an outpatient infertility clinic documented anxiety traits in 19.9% of participants [57]. Similarly, in a separate investigation encompassing 771 Chinese men with infertility, the prevalence rates for depression and anxiety were reported as 20.8% and 7.8%, respectively [58]. A Polish study comprising 188 infertile men revealed rates above cut-off points for depression and anxiety as 15.6% and 4.79%, respectively. In contrast, the observed prevalence rates in the Swedish and Italian samples were comparatively lower. The Swedish study reported major depression in 5.1% of males and various anxiety disorders in 4.9% of the sample [59], while the Italian study documented anxious symptoms in 4.5% of men and depressive symptoms in 6.9% of the cohort [60]. These discrepancies may stem from a multitude of factors, such as differences in sample sizes, variations in methodological approaches, and the utilization of diverse measurement instruments. Moreover, it is plausible to suggest that the reported psychopathological profiles are shaped by cultural nuances, as well as the ethnic and demographic contexts in which these studies were conducted. To gain a comprehensive understanding of these symptoms within specific population of infertile men, further research is essential to unravel the intricate underlying determinants. Although the literature indicates that men are more resilient to psychoemotional disturbances imposed by an infertility diagnosis and treatment than women [61], our findings are comparable with those observed using the same instrument, i.e., the DASS-42 questionnaire, among infertile females in Korea [62]. This may be contextualized by the notion documented in previous research highlighting the importance of gender assignment of the underlying cause of the conception difficulties. Namely, men’s reaction to fecundity issues approximates to that of women when couple infertility has been attributed to a male factor, regardless of the concomitant presence of a female factor [63]. 

As per the WHO definition, QoL refers to an individual’s subjective evaluation of their life, considering their expectations, objectives, standards, and concerns within the broader cultural and environmental context, societal framework, and personal value system. This holistic concept encompassing both affective and cognitive dimensions conveys the need for a departure from the mechanistic paradigm in contemporary medicine, in favor of integrating a humanistic element into healthcare practices [64,65]. Accordingly, the inclusion of psychosocial aspects alongside biomedical measures in addressing fertility issues has emerged as a pivotal factor in achieving favorable outcomes, as perceived by both clinicians and patients [66]. Score distributions across conceptual areas of the health-related QoL captured by the SF-36 questionnaire in our study exhibited a similar pattern to the findings reported for male partners in large cohorts of Italian [67] and Iranian [68] infertile couples undergoing in vitro fertilization treatment. Notable discrepancy between our study and the aforementioned research lies in the lower scores observed, specifically in the mental health domain within our cohort (Serbian sample: 49.00 ± 6.25 vs. Italian sample: 74.70 ± 15.60 and Iranian sample: 67.20 ± 17.80). Nevertheless, our observations align closely with the investigation conducted by Shindel et al. among American couples experiencing fertility challenges, where male participants demonstrated standardized scores with a mean value of 47.60 based on the mental health subscale of the SF-36 questionnaire [69]. Expectedly, inverse correlations were found between the SF-36 mental health score and those obtained from the DASS-42 depression (r = −0.320, *p* < 0.01), anxiety (r = −0.212, *p* < 0.05), and stress (r = −0.222, *p* > 0.05) subscales. Such convergence across employed instruments indicates consistency and supports reliability, thus enhancing confidence in the accuracy and the robustness of the observed findings. While subjective health profiles may not differ significantly between individuals with and without infertility, the duration of treatment appears to exert an influence on patients’ QoL [70]. However, reaching definitive conclusions in this regard remains elusive, as the existing literature presents certain contrasting findings, underscoring the intricate nature of the relationship between the infertility duration and its impact on overall well-being. In a study by Ragni et al., it was revealed that a longer period of struggling with infertility could have a detrimental effect on the physical functioning domain of the QoL [67]. Conversely, Rashidi et al. reported that neither the duration of infertility nor its underlying causes exerted a significant influence on the QoL [68]. Our cohort exhibited a statistically significant between-group difference in the role emotional concept of the SF-36, which provides valuable insights into how emotional problems may impact a person’s ability to fulfill their daily roles and responsibilities effectively. Specifically, patients with a longer treatment duration demonstrated lower scores in this domain, indicative of a less favorable emotional state. In line with expectations, inverse correlations were found between the SF-36 role emotional score and those obtained from the DASS-42 depression (r = −0.273, *p* < 0.01), anxiety (r = −0.350, *p* < 0.001), and stress (r = −0.246, *p* < 0.05) subscales (Figure 1). Furthermore, mediation analysis confirmed that the role emotional domain was directly affected by the explored infertility parameters and that the effect of the treatment duration was mediated by psychological distress expressed in the form of anxiety. The role emotional (RE) scale within the SF-36 questionnaire assesses a person’s perceived limitations in their daily activities or work due to emotional challenges. It explores three different aspects of these limitations: (1) if the individual felt compelled to reduce the time they spent on work or other regular activities as a result of emotional difficulties; (2) if the person felt that they achieved less in their work or activities than they desired due to emotional issues; (3) whether the individual noticed a decrease in the thoroughness or carefulness with which they carried out their work or activities because of emotional challenges. These findings suggest that extended periods of treatment may contribute to emotional challenges and impede individuals’ functioning in their daily lives. Continued research in this area is crucial for a comprehensive understanding of the underlying factors and mechanisms involved, ultimately guiding the development of effective interventions to enhance the QoL of those facing infertility challenges. Investigations conducted across various European countries have reported dropout rates ranging from 17% to 70% among couples undergoing assisted reproductive treatment [71], and it is estimated that in approximately 23% of cases, the premature termination may be attributed to the entailing emotional toll [72]. Hence, it might be prudent for infertility programs to integrate comprehensive psychological vulnerability assessments and establish supplementary counseling services as part of regular protocols, to alleviate psycho-emotional distress that hinders both patients’ QoL and treatment outcomes [73]. Enhancing resilience, as a valuable psychological asset, may serve as an effective strategy to mitigate the adverse consequences of psychoemotional disturbances related to infertility. Fostering resilience entails cultivating self-efficacy, developing effective problem-solving skills, and bolstering individuals’ ability to cope with the challenges posed by infertility. By harnessing these qualities, individuals are better equipped to navigate the emotional and psychological aspects of their fertility journey, potentially optimizing their overall well-being [74]. Theoretical studies and empirical findings suggest that the perception and interpretation patterns, as well as response trajectories in the reaction to health challenges, including infertility, are gender-specific. Considering the substantial discrepancy in both scientific and practical attention to psychosocial aspects of male infertility compared to the female position, there is a concern that men, known to exhibit a lower propensity for inquiring and engaging in medical consultations than women, may be disadvantaged in terms of receiving appropriate strategies for managing their emotional disturbances. Given the prevailing research emphasis on masculinity as a determinant of men’s engagement with medical assistance, the exploration of male needs and the quality of support they receive throughout the illness continuum remains relatively limited, accentuating the necessity for a more comprehensive understanding of these dynamics [75]. 

Although extensive measures were undertaken to mitigate bias and ensure meticulous data collection and analysis, it is crucial to acknowledge the presence of certain limitations within this study. A noteworthy constraint stems from the implementation of a cross-sectional design, which inherently impedes the establishment of the precise temporal sequencing of events and consequently precludes the derivation of causal inferences. While the comprehensive diagnostic process involved a careful examination of the medical and reproductive history, a thorough physical examination with particular attention to secondary sexual characteristics and genitalia, as well as semen analysis, followed by second-level examinations, including hormonal evaluations and microbiological exams, we made a deliberate choice to primarily focus on semen parameters as the cornerstone of male fertility assessments. Consequently, the detailed elaboration of other factors remained beyond the scope of the present article. Due to the lack of baseline data on the QoL and symptoms of psycho-emotional disturbances among the studied individuals before the diagnosis of infertility, the potential for protopathic bias cannot be entirely disregarded. The single-center nature of this research and limited sample size may restrict the generalizability of the findings beyond the specific setting under investigation. The recruitment of men from a specific participant pool within a particular clinical facility raises caution regarding the broader applicability of the results to diverse populations or alternative healthcare contexts. Notwithstanding the potential impact of selection bias, the present study offers relevant evidence regarding the characteristics and experiences of infertile men seeking treatment at a major urology clinic in Serbia, thus contributing to the scarce knowledge base in this filed. Although the utilization of self-reported data brings attention to the potential influence of recall and/or social-desirability bias, proactive measures were made to alleviate their impact. These include the application of validated instruments, standardized investigation procedures, a clear indication of instructions provided by well-trained medical professionals, and the maintenance of a non-judgmental and supportive stance throughout the data collection process, fostering an atmosphere of trust and openness. The inclusion of diverse data sources, such as medical records and clinician assessments, further complemented the self-report data, providing a more comprehensive and objective perspective. Such an approach enhanced the robustness of our study and contributed to a more accurate understanding of the complex factors involved in male infertility and its impact on the QoL.

## 5. Conclusions

While it is imperative to conduct further longitudinal and multicentric research to validate and expand upon our findings, the present study offers valuable insights into the intricacies of QoL and psycho-emotional disturbances experienced by men undergoing infertility treatment in Serbia. By delving into the multidimensional nature of the distress faced by these individuals, our research contributes to the limited body of knowledge in this area, highlighting the necessity for comprehensive support strategies that holistically address their unique needs. These findings may serve as a fundamental platform for future investigations, enabling a deeper understanding of the complex dynamics between physical and psycho-emotional factors within the context of male infertility. Such advancements hold great potential for the development of targeted interventions and the delivery of enhanced patient care, ultimately striving towards improved outcomes and well-being for these individuals.

## Figures and Tables

**Table 1:** Study sample overview.

Characteristics	Patients Treated for Infertility < 2 Years; n = 50	Patients Treated for Infertility ≥ 2 Years; n = 46	*p* ^1^	Total Sample n = 96
**Age, years**, $\bar{X}$ **± SD**	36.74 ± 5.56	38.72 ± 5.78	0.193	37.69 ± 5.72
**Body Mass Index (BMI), kg/m^2^**, $\bar{X}$ **± SD**	27.60 ± 4.50	27.85 ± 3.45	0.331	27.72 ± 4.01
Highest level of formal education *, n (%)				
ISCED 0/1: Less than primary/primary education	4 (8.0)	1 (2.17)	0.279	5 (5.2)
ISCED 2/3: Lower/upper secondary education	22 (44.0)	26 (56.52)		48 (50.0)
ISCED 4–8: Post-secondary non-tertiary/tertiary education **	24 (48.0)	19 (41.30)		43 (44.8)
Occupational profile ***, n (%)				
Unemployed	1 (2.0)	1 (2.2)	0.915	2 (2.1)
Skilled agricultural, forestry, and fishery workers	1 (2.0)	0 (0.0)		1 (1.0)
Elementary occupations	5 (10.0)	3 (6.5)		8 (8.3)
Plant and machine operators, assemblers/Craft workers	8 (16.0)	7 (15.2)		15 (15.6)
Technicians/Clerical support/Service and sales workers	19 (38.0)	21 (45.6)		40 (41.7)
Professionals/Managers	10 (20.0)	9 (19.6)		19 (19.8)
Other	6 (12.0)	5 (10.9)		11 (11.5)
**Residential region, n (%)**				
Belgrade (capital city) region	40 (80.0)	39 (84.8)	0.539	79 (82.3)
Other geographical regions	10 (20.0)	7 (15.1)		17 (17.7)
**Self-reported socio-economic status, n (%)**				
High	7 (14.0)	7 (15.2)	0.412	14 (14.6)
Middle	41 (82.0)	34 (73.9)		75 (78.1)
Low	2 (4.0)	5 (10.9)		7 (7.3)
**Currently smoking, n (%)**				
Yes	18 (36.0)	10 (21.7)	0.125	28 (29.2)
No	32 (64.0)	36 (78.3)		68 (70.8)
**Alcohol consumption, n (%)**				
Yes	28 (56.0)	26 (56.5)	0.959	54 (56.3)
No	22 (44.0)	20 (43.5)		42 (43.8)
**History of sexually transmitted diseases, n (%)**				
Yes	9 (18.0)	4 (8.7)	0.183	13 (15.5)
No	41 (82.0)	42 (91.3)		83 (86.5)
**History of urological surgery, n (%)**				
Yes	8 (16.0)	7 (15.2)	0.916	15 (15.6)
No	42 (84.0)	39 (84.8)		81 (84.4)
**Having at least one child, n (%)**				
Yes	10 (20.0)	4 (8.7)	0.117	14 (14.6)
No	40 (80.0)	42 (91.3)		82 (85.4)
**Duration of infertility treatment, months**, $\bar{X}$ **± SD**	13.90 ± 2.86	57.52 ± 36.91	NA	34.80 ± 33.61

* ISCED, International Standard Classification of Education; ** Tertiary education includes short-cycle tertiary education and Bachelor’s/Master’s and Doctoral or equivalent level; *** occupational profile categories were defined based on The International Classification of Occupations (ISCO); ^1^ Chi square test, except for age and BMI (Man-Whitney U-test); NA—not applicable.

**Table 2:** Stratification of patients based on the treatment duration and qualitative and quantitative semen analysis.

Semen Analysis Category	Criteria	Patients Treated for Infertility < 2 Years; N = 50 n (%)	Patients Treated for Infertility ≥ 2 Years; N = 46 n (%)	*p* *	Total Sample; N = 96 n (%)
**Normozoospermia**	Sperm concentration exceeding the lower reference limit of 15 × 10^6^ spermatozoa/mL of ejaculate	23 (46.0)	18 (39.1)	0.497	41 (42.7)
**Azoospermia**	Absence of spermatozoa in the ejaculate	7 (14.0)	3 (6.5)	0.230	10 (10.4)
**Oligozoospermia**	Sperm concentration below the lower reference limit of 15 × 10^6^ spermatozoa/mL of ejaculate	20 (40.0)	25 (54.4)	0.159	45 (46.9)
Mild	10–15 × 10^6^ spermatozoa/mL of ejaculate	3 (6.0)	2 (4.4)	0.171	5 (5.2)
Moderate	5–10 × 10^6^ spermatozoa/mL of ejaculate	9 (18.0)	6 (13.0)		15 (15.6)
Severe	<5 × 10^6^ spermatozoa/mL of ejaculate	8 (16.0)	17 (36.9)		25 (26.0)
**Asthenospermia**	<40% total sperm motility or <32% rapid progressive motility	8 (16.0)	23 (50.0)	<0.001	31 (32.3)
**Teratozoospermia**	<4% of morphologically normal spermatozoa	13 (26.0)	20 (43.5)	0.072	33 (34.4)
**Pyospermia**	>1.0 × 10^6^ leucocytes per mL of ejaculate	4 (8.0)	5 (10.9)	0.630	9 (9.4)
**Semen volume**					
Hypospermia	<1.5 mL	5 (10.0)	6 (13.0)	0.703	11 (11.5)
Normospermia	1.5–6.0 mL	41 (82.0)	38 (82.6)		79 (82.3)
Hyperspermia	>6.0 mL	4 (8.0)	2 (4.4)		6 (6.3)

* Chi square test.

**Table 3:** Evaluation of psychosocial distress among men undergoing infertility treatment based on The Depression Anxiety Stress Scale (DASS-42).

Psychosocial Distress Domain Based on DASS-42*	Scale Score			*p* ^1^	Bayes Factor (BF_10_)	Conventional Severity Categories, n (%)				
	$\bar{X}$ ± SD	Median	IQR			Normal	Mild	Moderate	Severe	Extremely Severe
**Depression**				0.116	0.582					
Patients treated for infertility < 2 years; n = 50	3.76 ± 4.38	2.50	1.00–5.00			45 (46.9)	3 (3.1)	2 (2.1)	-	-
Patients treated for infertility ≥ 2 years; n = 46	5.34 ± 5.48	4.00	1.25–7.00			38 (39.5)	4 (4.2)	2 (2.1)	2 (2.1)	-
Total sample, n = 96	4.52 ± 4.98	3.00	1.00–6.00			83 (86.5)	7 (7.3)	4 (4.2)	2 (2.1)	-
**Anxiety**				0.605	0.244					
Patients treated for infertility < 2 years; n = 50	4.12 ± 4.94	3.00	1.00–5.00			42 (43.8)	3 (3.1)	3 (3.1)	1 (1.0)	1 (1.0)
Patients treated for infertility ≥ 2 years; n = 46	3.63 ± 3.09	2.50	2.00–5.00			43 (44.8)	1 (1.0)	1 (1.0)	1 (1.0)	-
Total sample, n = 96	3.89 ± 4.15	3.00	1.00–5.00			85 (88.5)	4 (4.2)	4 (4.2)	2 (2.1)	1 (1.0)
**Stress**				0.364	0.261					
Patients treated for infertility < 2 years; n = 50	10.36 ± 8.11	9.00	4.25–13.75			39 (40.6)	3 (3.1)	4 (4.2)	3 (3.1)	1 (1.0)
Patients treated for infertility ≥ 2 years; n = 46	11.24 ± 7.59	11.00	6.00–14.00			35 (36.5)	4 (4.2)	5 (5.2)	2 (2.1)	-
Total sample, n = 96	10.78 ± 7.83	10.00	5.00–14.00			74 (77.1)	7 (7.3)	9 (9.4)	5 (5.2)	1 (1.0)

^1^ Mann–Whitney U-test, IQR, interquartile range.

**Table 4:** Health-related quality of life among men undergoing infertility treatment itemized according to the 36-Item Short Form Health Survey questionnaire (SF-36) domains.

Health-Related Quality of Life Domain Based on SF-36	Total Sample, n = 96			Patients Treated for Infertility < 2 Years; n = 50			Patients Treated for Infertility ≥ 2 Years; n = 46			*p* ^1^	Bayes Factor (BF_10_)
	$\bar{X}$ ± SD	Median	IQR	$\bar{X}$ ± SD	Median	IQR	$\bar{X}$ ± SD	Median	IQR		
**Physical functioning**	90.16 ± 17.75	95.00	90.00–100.00	90.30 ± 17.24	95.00	86.20–100.00	90.00 ± 18.47	95.00	90.00–100.00	0.676	0.245
**Role physical**	87.50 ± 26.66	90.00	85.00–100.00	89.50 ± 22.07	90.00	85.00–100.00	85.33 ± 30.99	90.00	85.00–100.00	0.750	0.285
**Bodily pain**	75.34 ± 21.68	87.00	62.00–90.00	77.34 ± 21.61	90.00	74.00–90.00	73.17 ± 21.78	84.00	62.00–90.00	0.146	0.375
**General health**	74.67 ± 17.24	77.00	64.25–87.00	72.76 ± 17.85	77.00	62.75–84.25	76.74 ± 16.49	80.00	65.50–89.25	0.317	0.408
**Vitality**	65.42 ± 11.14	65.00	60.00–75.00	66.80 ± 10.14	65.00	60.00–75.00	63.91 ± 12.06	62.50	55.00–75.00	0.230	0.424
**Social functioning**	83.59 ± 17.07	87.50	75.00–100.00	83.25 ± 18.49	87.50	75.00–100.00	83.96 ± 17.81	87.50	75.00–100.00	0.893	0.227
**Role emotional**	84.03 ± 30.58	87.50	66.67–100.00	80.00 ± 24.51	87.50	75.00–100.00	77.54 ± 35.18	87.50	66.67–100.00	0.050	0.569
**Mental health**	49.00 ± 6.25	48.00	44.00–52.00	49.36 ± 5.58	50.00	45.00–52.00	48.61 ± 6.96	48.00	44.00–55.00	0.352	0.262

^1^ Mann–Whitney U-test, IQR, interquartile range.

## Data Availability

The data supporting reported results can be found upon request in the form of datasets available at the Clinic of Urology, University Clinical Centre of Serbia.
